# R software package based statistical optimization of process components to simultaneously enhance the bacterial growth, laccase production and textile dye decolorization with cytotoxicity study

**DOI:** 10.1371/journal.pone.0195795

**Published:** 2018-05-02

**Authors:** Sunil Bhavsar, Pravin Dudhagara, Shantilal Tank

**Affiliations:** 1 Department of Biosciences (UGC-SAP-II), Veer Narmad South Gujarat University, Surat, Gujarat, INDIA; 2 Bioinformatics and Supercomputer Laboratory, Department of Biosciences (UGC-SAP-II), Veer Narmad South Gujarat University, Surat, Gujarat, INDIA; Universidad Nacional Autonoma de Mexico Centro de Nanociencias y Nanotecnologia, MEXICO

## Abstract

The thermophilic bacterium, *Bacillus licheniformis* U1 is used for the optimization of bacterial growth (R1), laccase production (R2) and synthetic disperse blue DBR textile dye decolorization (R3) in the present study. Preliminary optimization has been performed by one variable at time (OVAT) approach using four media components viz., dye concentration, copper sulphate concentration, pH, and inoculum size. Based on OVAT result further statistical optimization of R1, R2 and R3 performed by Box–Behnken design (BBD) using response surface methodology (RSM) in R software with R Commander package. The total 29 experimental runs conducted in the experimental design study towards the construction of a quadratic model. The model indicated that dye concentration 110 ppm, copper sulphate 0.2 mM, pH 7.5 and inoculum size 6% v/v were found to be optimum to maximize the laccase production and bacterial growth. Whereas, maximum dye decolorization achieved in media containing dye concentration 110 ppm, copper sulphate 0.6 mM, pH 6 and inoculum size 6% v/v. R package predicted R^2^ of R1, R2 and R3 were 0.9917, 0.9831 and 0.9703 respectively; likened to Design-Expert (Stat-Ease) (DOE) predicted R^2^ of R1, R2, and R3 were 0.9893, 0.9822 and 0.8442 respectively. The values obtained by R software were more precise, reliable and reproducible, compared to the DOE model. The laccase production was 1.80 fold increased, and 2.24 fold enhancement in dye decolorization was achieved using optimized medium than initial experiments. Moreover, the laccase-treated sample demonstrated the less cytotoxic effect on L132 and MCF-7 cell lines compared to untreated sample using MTT assay. Higher cell viability and lower cytotoxicity observed in a laccase-treated sample suggest the impending application of bacterial laccase in the reduction of toxicity of dye to design rapid biodegradation process.

## Introduction

Synthetic dyes and dyestuff extensively use in textile, paper, cosmetic and pharmaceutical industries. Textile industries in developing countries showed a significant increase in the use of synthetic organic dyes as the coloring agent. Approximately 10–15% of the dyes release into the environment during manufacturing process and usage [1]. Nearly, 30–60 g dyestuff along with 70–150 L water are required to impart the colour in 1 kg of cotton; the wastewater produced or the process has 20–30% of the applied unfixed reactive dyes, with an average up to a concentration of almost 2000 ppm and dyeing auxiliaries [2]. So, the waste water of textile manufacturing unit contains a variety of organic pollutants, among them synthetic dyes are chief pollutant [3]. Worldwide, 280,000 tonnes of textile dyes are discharged in industrial effluents every year [4] and cause detrimental effects on environment and health. Dyes include a broad spectrum of various chemical structures, based on substituted aromatic and heterocyclic groups such as an aromatic amine (C_6_H_5_–NH_2_), phenyl (C_6_H_5_–CH_2_) and naphthyl (NO_2_–OH). A large number of dyes are azo compounds (–N–N–), which are linked by an azo bridge [5]. In anaerobic environments, azo linkages easily break down and act as an electron acceptor for reduced flavin nucleotides, and reduction increases with redox mediators [6–9]. Anaerobic azo dye reduction resulting in the formation of toxic aromatic amines which are not mineralized anaerobically [10], except a few toxic aromatic amines substituted with hydroxyl and carboxyl groups degrade under methanogen conditions [11]. In contrast to the anaerobic conditions, aromatic amines easily degrade in aerobic condition [12]. However, azo dyes are resistant to bacterial attack under aerobic condition [13–17] because the presence of oxygen usually inhibits azo bond reduction activity. Some selected aerobic bacterial strains possess the ability to reduce the azo linkage with the help of oxygen catalysed by aerobic azoreductases and produced aeromatic amines [18–20]. Numerous review articles have summarised the studies on aerobic bacterial azo dye reduction [21–29]. Bacterial azo dye biodegradation proceeds in two phases. The first step takes in reductive cleavage of the dyes azo linkages, resulting in the formation of usually colourless but potentially hazardous aromatic amines remains which degraded in a second step [30].

The biodegradation mechanism of xenobiotics containing aromatic amines using the microbial system accomplished through the action of the biotransformation enzymes. Several reports demonstrated the biodegradation of such complex organic substances by enzymatic mechanisms, using laccase [31], lignin peroxidase [32], nicotinamide adenine dinucleotide dehydrogenase—Dichlorophenolindophenol (NADH-DCIP) reductase [33], tyrosinase [34], hexane oxidase [35] and aminopyrine N-demethylase [36]. These enzymes decolorize azo dyes without direct cleavage of the azo bonds through a highly non-specific free radical mechanism and thereby avoiding the formation of toxic aromatic amines [37]. However, in the complete degradation or decolorization of dye, the cocktail of enzymes are simultaneously produced by the bacteria. Higher production of multiple enzymes like azoreductase, lignin peroxidase, and laccase are involved in the Methyl Orange decolorization [38]. Among these enzymes, laccase widely reported due to their broad substrate specificity.

Many traditional physicochemical processes are available for the removal of color and pollutants present in the textile wastewater. Adsorption, chemical oxidation, and reduction, chemical precipitation, flocculation, and photolysis are few conventional methods commonly used for the decolorization of dye containing wastewater [39–41]. These methods are mostly ineffective, expensive and produced colossal sludge and by-products. Instead of these traditional methods, bacterial enzymatic degradation or decolorization have several advantages, including (i) environmentally-friendly process, (ii) being cost competitive, (iii) producing less toxic sludge, (vi) yielding end products that are non-toxic or have complete mineralization; and (v) requiring less water consumption compared to physicochemical methods [21,42]. However, the effectiveness of microbial mediated dye decolorization is a challenging task because the process is susceptible to various chemical and physical parameters moreover the successful decolorization depende on the adaptability and the metabolic activity of the selected microorganisms.

The most important aspects of microbial decolorization are the formulation of the appropriate process medium that favors the growth, enzyme production, and dye decolorization. So, the optimization of the process parameters become essential to design an effective biodecolorization strategy. Traditional OVAT approach to scaling up the media parameters for responses by maintaining other involved variables at an unspecified constant level do not show the pooled effect of all the involved variables. Furthermore, the OVAT method is time-consuming and requires more experiments to determine optimum levels for each parameter. Statistical experimental design can overcome these limitations of the classical method and convenient tool for optimizing production media with significant impact on enzyme production as it can provide statistical models, which aid in understanding the interactions among the media parameters at varying levels [43]. Response Surface Methodology (RSM) is an effective optimization tool which allows simultaneous evaluation of the factors confirmed and their interactions can identify with fewer experimental trials [44]. Box–Behnken design (BBD) is amongst the most commonly used methodology in various process optimization experiments. BBD regression model provided an excellent explanation of the relationship between the independent variables and the response [45]. The most of commercial softwares, i.e., DOE (Stat-Ease, Minneapolis, United States of America), JMP (SAS, Cary, North Carolina) and Minitab are usually expensive and proprietary, whereas; R is an open source software which allows execution of various statistical techniques and can extend via different packages. So, it offers a broad range of statistical and graphical techniques with more updated packages. Statistical design of experiments embedded in R open source software by R-Commander (Rcmdr) package with RcmdrPlugin.DoE plugin [46,47]. The package provides a platform-independent Graphical User Interface (GUI) to design experiments. Statistical design functionality can access through the menu design that added to the Rcmdr menu. In the present research, DOE and R package compared mainly for modelling perspective. To the best of our knowledge, there are no reports on the use of statistical design of R package for optimization of three responses simultaneously. We also emphasized R software package’s sensitivity analysis and its usefulness in the optimization process through the validation approach.

This study aims to optimize the medium composition mainly required for growth of isolate, the laccase production and dye decolorization by a *Bacillus licheniformis* U1 strain using R open source software with R Commander (Rcmdr) package.

## Materials and methods

### Chemicals

**Fig 1:**
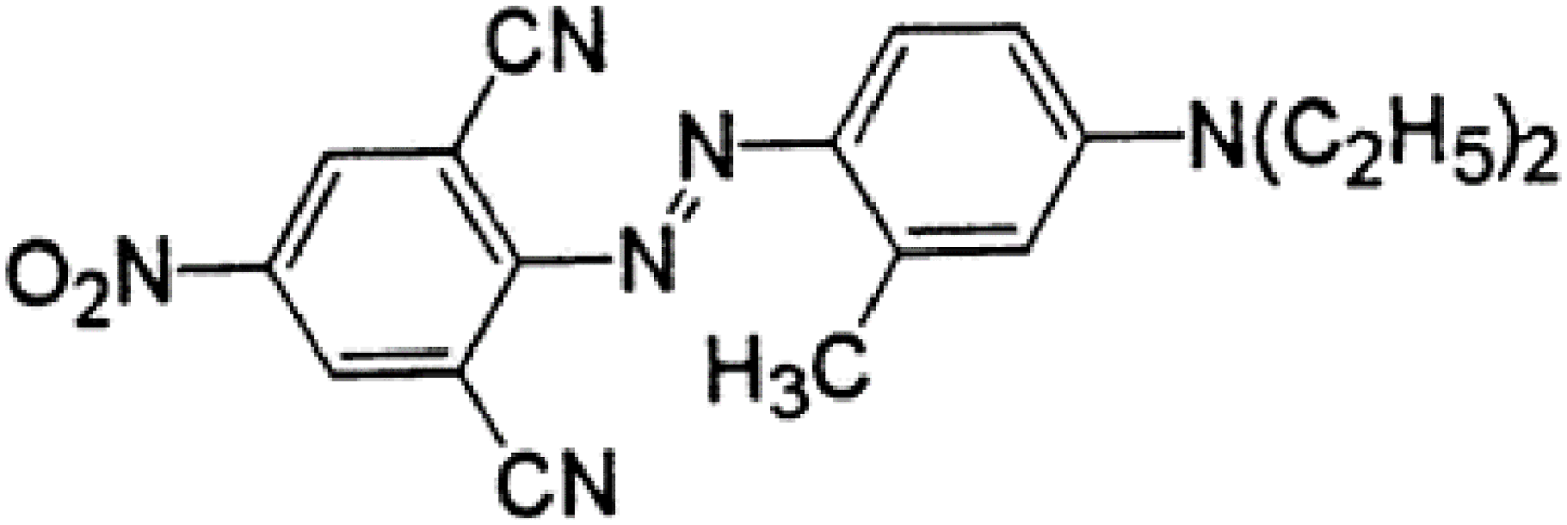
Chemical structure of disperse blue DBR.

Syringaldazine (N, N’-bis (3, 5- dimethoxy-4-hydroxybenzylidene hydrazine)) was purchased from Sigma-Aldrich (St. Louis, MO, USA). Disperse Blue DBR (C.I. Disperse Blue 366) (Fig 1) acquired from the famous textile market of Surat, Gujarat, India. All other chemicals and reagents were of analytical grade. Cell lines obtained from National Centre for Cell Science (NCCS), Pune. India.

### Source of microorganism

Bacterial strain initially isolated in gelatine casein medium as a potent protease producer in our laboratory from the Unnai hot spring water sample, Unnai, South Gujarat. The strain was identified based on partial gene sequence of 16S rRNA, and the sequence submitted to GenBank (Accession No. GU979026) [48]. The isolate maintained on nutrient agar, pH 8.0 and subculture after every 30 days on same solid media.

### Screening of isolate for laccase activity

A nutrient agar plate containing 0.216mM of syringaldazine (prepared 0.1 M phosphate buffer, pH 6.5) as a substrate was used to test the laccase production by isolates. A well bored into the solid agar plate supplemented with syringaldazine followed by pouring 100μl of overnight active culture (A_620_ = 0.82). The plate was incubated in an incubator at 40°C for three days, and visual observation was carried out for substrate utilization surrounding bore to confirm the qualitative screening of laccase activity.

### Laccase production and activity assay

The time course of growth and the laccase production studied in Bushnell-Haas (BH) medium supplemented with 200ppm dye and 0.2% w/v yeast extract and inoculated 10% v/v overnight grown culture (A_620_ = 0.82) followed by incubation at 40°C in shaking condition at 150 rpm. Periodically, the sample was withdrawn aseptically to test the growth at A_620_ nm, and culture was harvested by centrifugation at 8000 rpm for 10 min at 4°C. The cell-free extract was used as a crude preparation of enzyme to measure laccase activity.

The crude enzyme activity was estimated by the Ride’s method using syringaldazine as a substrate [49]. Exactly 0.5 mL of crude enzyme was added to 2.2 mL of 0.1 M potassium phosphate buffer (pH 6.5) and parallel blank reaction performed by adding 0.5 mL distilled water instead of the enzyme in 0.1 M phosphate buffer (pH 6.5). After proper mixing, 0.3 mL of 0.216mM Syringaldazine added and again mixes by inversion and record the increase in A_530_ nm for approximately 10 minutes. The Δ530 nm/min was obtained using the maximum linear rate for both the test and blank. The enzyme activity was calculated by formula 1.

$$\text{Enzyme}\;\text{activity}\;\left(\text{Units}/\text{mL}\right)=\frac{\text{A}530\frac{\text{nm}}{\text{min}}\text{Test}\;-\;\text{A}530\frac{\text{nm}}{\text{min}}\text{Blank}\;\times \;\left(\text{df}\right)\;}{\left(0.001\right)\;\times \;\left(0.5\right)}$$ (1)

Where, df = dilution factor, 0.001 = the change in A_530_ nm/min. per unit of laccase at pH 6.5 at 30°C in a 3 mL reaction mix and 0.5 = volume (mL) of enzyme used.

### Optimization of growth, laccase production and dye decolorization by OVAT approach

The OVAT approach used for preliminary screening of various media components influencing the growth of isolate, laccase production, and dye decolorization. The culture was grown in 250 mL Erlenmeyer flask containing 50 mL BH medium supplemented with 0.2% yeast extract with variable dye concentration (20–200 ppm), copper sulphate solution (0.2–1 mM), pH (6–9), and inoculum size (2–6% v/v) incubate at 40°C, 150 rpm for 24 hrs. The medium without inoculation considered as a control. The difference in growth measured at A_620_ nm. Laccase enzyme activity was calculated by Ride’s method as described in an earlier section and dye decolorization was measured at A_614_ nm using a spectrophotometer (UV-1800, Shimadzu). Dye decolorization experiment was carried out using textile dye disperse blue DBR (λmax = 614 nm). Reactions initiated by the addition of bacterial laccase and incubated at 40°C and 150 rpm. Control sample runs in parallel without the addition of bacterial laccase. Percent of dye decolorization was calculated as the formula 2.

Decolorization(%)=[(Ai−At)/Ai]×100 (2)

Where Ai: initial absorbance of the dye; At final absorbance of the dye at 10 min time interval [50].

### Optimization and validation of media

The media components parameters profoundly influence the growth of isolates (R1), laccase activity (R2), and dye decolorization (R3). So, to maximize, these responses, four process variables, i.e., dye concentration (X_1_), copper sulphate (X_2_), pH (X_3_) and inoculum size (X_4_) were used for optimization by Box-Behnken design (BBD) using R package and compared with DOE.

#### Box-Behnken design for optimization

After observing the preliminary result of OVAT experiments, four independent variables such as X_1_, X_2_, X_3,_ and X_4_ were identified to test their effect on responses R1, R2, and R3 using BBD [51]. Each selected variable studied at three levels- low, medium and high coded as −1, 0 and +1 in a total of 29 experiments. The value of variables coded according to the following Eq 3.

$$\;x_{i}=\frac{\text{Xi}-\text{Xo}}{\Delta \text{X}}\;\text{i}=1,2,3,4$$ (3)

Where ***x***_***i***_ is the coded value of an independent variable; Xi is the actual value of an independent variable; Xo is the actual value of an independent variable at centre point; and ΔX is the step change value of an independent variable. Each selected variable was studied at three levels- low, medium, and high. All the three responses corresponding to the combined effects of four variables were studied in their specified ranges as shown in Table 1.

**Table 1:** Levels and code of variables chosen for the Box-Behnken design.

Variables	Range and value of variables		
	-1	0	1
**X_1_: Dye Concentration (ppm)**	20	110	200
**X_2_: Copper Sulfate (mM)**	0.2	0.6	1
**X_3_: pH**	6	7.5	9
**X_4_: Inoculum Size (% v/v)**	2	4	6

The role of each variable, their interactions, and statistical analyses in obtaining predicted yields explained by applying the following second order polynomial quadratic model Eq 4.

$$\begin{array}{l} \text{R}1=\beta_{0}+\sum \beta_{1}{\text{X}}_{1}+\sum \beta_{2}{\text{X}}_{2}+\sum \beta_{3}{\text{X}}_{3}+\sum \beta_{4}{\text{X}}_{4}+\sum \beta_{12}{\text{X}}_{1}{\text{X}}_{2}+\sum \beta_{13}{\text{X}}_{1}{\text{X}}_{3}+\sum \beta_{14}{\text{X}}_{1}{\text{X}}_{4}+\sum \beta_{23}{\text{X}}_{2}{\text{X}}_{3}\\ +\sum \beta_{24}{\text{X}}_{2}{\text{X}}_{4}+\sum \beta_{34}{\text{X}}_{3}{\text{X}}_{4}+\sum \beta_{11}{\text{X}}_{1}{}^{2}+\sum \beta_{22}{\text{X}}_{2}{}^{2}+\sum \beta_{33}{\text{X}}_{3}{}^{2}+\sum \beta_{44}{\text{X}}_{4}{}^{2} \end{array}$$ (4)

Where, R1 is the predicted responses, β_0_ offset term (Intercept process effect), β_1_, β_2_, β_3,_ and β_4_ are linear effects; β_12_, β_13_, β_14_, β_23,_ β_24_ and β_34_ are cross product effects, and β_11_, β_22_, β_33_ and β_44_ are squared effects. The interactions are represented by X_1_X_2_, X_1_X_3_, X_1_X_4_, X_2_X_3_, X_2_X_4_, and X_3_X_4_. This equation represents the quadratic effect of X_1_, X_2_, X_3,_ and X_4_. Similar equations obtained for other responses R2 and R3.The statistical design performed in R open source software with RcmdrPlugin.DoE plugin and also carried out in DOE (Academic version 9.0) for comparison of model’s goodness of fit.

#### Validation of model

The validity of the chosen polynomial quadratic model, predicted by R software’s Rcmdr package was confirmed experimentally with triplicate experiments.

### Cytotoxicity evaluation of decolorized dye

The cellular toxicity of laccase-treated and untreated dye samples was evaluated using the MTT assay [52,53]. This test evaluated the effect of the laccase-treated dye for the cell viability percentage of two cell lines, i.e., L132 (Human normal lung epithelial) cell line and MCF-7 (breast cancer cell line). The cytotoxicity measured by comparing the absorbance of each well was recorded at 570nm using a multimode microplate reader (Epoch, BioTek Instrument Ltd). The untreated cells used as a control for calculating the relative percentage cell viability and cell toxicity from the following Eqs 5 and 6.

$$\%\;Cell\;Viability=\frac{\text{A}570\;\text{of}\;\text{TS}}{\text{A}570\;\text{of}\;\text{CS}}\times 100\%$$ (5)

Where, TS is treated sample, and CS is a control sample.

%Celltoxicity=100−%CellViability (6)

## Results and discussion

### Source of bacterial strain and identification

Spore-forming-rod-shaped, the Gram-positive bacterium was isolated from the Unnai hot spring (N 20° 51’ 14.1091" and E 73° 20’ 9.4986"), Gujarat, India. The isolate previously identified as a *Bacillus licheniformis* U1 [48]. The hot spring is natural thermal habitat, that harbour diverse thermophilic bacteria and a potential source of thermostable laccase [54,55]. The use of thermophilic laccase for dye degradation is worth exploring because the dye waste release from textile industries are usually warm. So, the elevated temperature of waste is suitable for the catalysis process of laccase. The use of thermophilic laccase is a vital component to establish bio-based industrial process development, especially for textile industries.

### Screening of isolate for laccase enzyme and initial enzyme production

**Fig 2:**
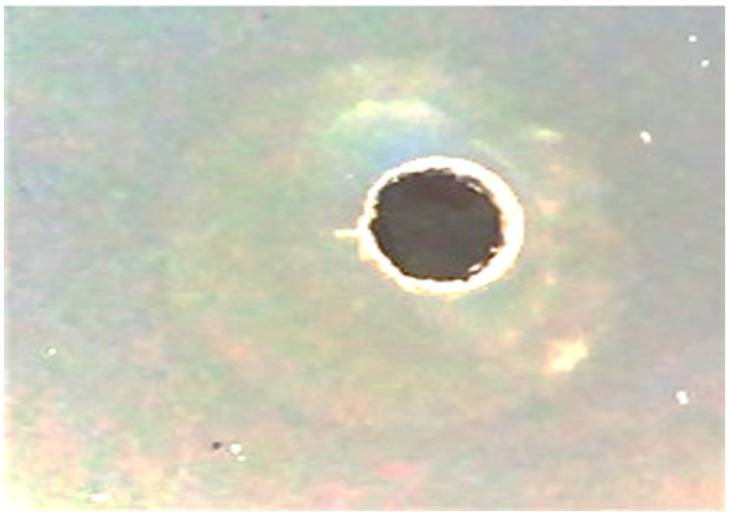
Zone of syringaldazine oxidation by laccase.

Syringaldazine supplemented nutrient agar plate indicating the halo zone surrounding the well poured with active culture suggests an ability of an isolate to oxidise syringaldazine by extracellular laccase production (Fig 2). Syringaldazine considered as a unique substrate for laccase screening through free radical generation [56]. *B*. *licheniformis* U1 has shown maximum 186.54 U/mL laccase and dye decolorization was 22.85% after 76 hrs. However, prolonged incubation reduced the laccase activity which is due to degradation of laccase by an extracellular protease.

### OVAT optimization of media

Preliminary studies with selected variables (i.e., X_1_, X_2_, X_3_ and X_4_) using OVAT approach shown to be important, and found to be persuasive in regulating all responses included in the study (Table 2). The optimum concentration of all these four factors is essential to design the biodecolorization of dye, the detail justification given here. Bacteria do not readily utilized the azo dye and often required the additional organic carbon source to promote the dye degradation [28]. However, bacteria can grow on azo compounds as a sole carbon source by cleavage of–N = N–bonds reductively and utilise amines as the source of carbon and energy for their growth. McMullan et al. (2001) reported the aerobic growth of *Xenophilus azovorans* KF 46 and *Pigmentiphaga kullae* K24 using carboxy orange I and carboxy orange II, respectively [27]. The high tolerance of dye is one of the chief criteria for growth promotion of isolate, induction of laccase and dye decolorization [57]. The luxurious growth of isolate at 6.0 to 8.0 pH in the presence of copper sulphate, dye, and proper inoculum size is the crucial feature for the mass production of the enzyme. The pH has a significant effect on the efficiency of dye decolorization. The optimum pH for laccase in bacillus species including *Bacillus tequilensis* SN4, *Bacillus subtilis*, and γ -Proteobacterium JB reported as being in the range of 7.0 to 8.0. The neutral pH usually favors the growth and laccase production in *Bacillus sp*.[58].

**Table 2:** Optimum values of OVAT preliminary optimization.

Parameters (range)	Optimum values		
	R1	R2	R3
**Dye Concentration (20–200 ppm)** (With an interval of 20 ppm)	120	120	100
**Copper Sulphate (0.2–1.0 mM)** (With an interval of 0.2 mM)	0.4	0.4	0.6
**pH (6.0–9.0)** (With an interval of 0.5 pH)	7.0	7.0	7.0
**Inoculum Size (2.0–6.0%) v/v** (With an interval of 1.0% v/v)	4.0	3.0	4.0

Laccases are copper-containing oxidases (type-1) and show a role in copper tolerance in certain bacterial species [59]. Copper sulphate acted as an inducer of laccase activity and growth of isolate too [60]. Therefore, the addition of copper ion as a medium component was used to enhance laccase production. Inoculum size plays a major role in enzyme production. A lower level of inoculum may not be sufficient to initiate growth, whereas a higher level may cause competitive inhibition [61]. The optimum results of various responses obtained in the presence of 3.0–4.0% v/v of inoculum are subtly lower indicate the economically feasible volume. Many researchers have reported different inoculum sizes for the optimum growth of the various bacteria viz., (i) 0.3% v/v for *Bacillus tequilensis* SN4 [62], (ii) 4.0% v/v for *Serratia marcescens* [63], (iii) 10% v/v for Bacillus sp PK4 [64] and (iv) 15% v/v for *Streptomyces psammoticus* [65]. However, the inoculum size is varies depending on the species of bacteria for their optimum growth.

Based on the OVAT result, RSM study conducted on selected variables for optimization of bacterial growth, laccase production, and dye decolorization.

### RSM optimization of media

#### Box-Behnken design for optimization

A total of 29 sets of experiments performed with different combinations of variables X_1_, X_2_, X_3_ and X_4_ using BBD which also includes five centre point of second order response surface (Table 1). This centre point provided process stability and intrinsic variability. The actual and predicted values of all responses by R and DOE are comparable. R based predicted values of all three responses are closer to respective experimental values than DOE based predicted values. It suggests precise reliability of R than DOE (Table 3). The regression coefficient for linear terms X_1_, X_2_, X_3_, and X_4_ were highly significant (p ≤ 0.001) for responses R1 and R2. Except for X1, all other linear terms were highly significant (p ≤ 0.001) for response R3. The most of interaction of each linear term was also significant for all responses (Table 4). Student t-test also performed for the significance of the regression coefficients of the parameters. The fitted polynomial quadratic response surface model is as specified by Eqs (7)–(9) for responses R1, R2, and R3 respectively.

$$\begin{array}{l} R1=\;0.146\;+\;0.034{\text{X}}_{\text{1}}\;-\;0.075{\text{X}}_{\text{2}}\;-\;0.021{\text{X}}_{\text{3}}\;+\;0.041{\text{X}}_{\text{4}}\;+\;0.045{\text{X}}_{\text{1}}{\text{X}}_{\text{2}}\;+\;0.001{\text{X}}_{\text{1}}{\text{X}}_{\text{3}}\;+\;0.049{\text{X}}_{\text{1}}{\text{X}}_{\text{4}}\;-\\ 0.033{\text{X}}_{\text{2}}{\text{X}}_{\text{3}}\;-\;0.099{\text{X}}_{\text{2}}{\text{X}}_{\text{4}}\;+\;0.024{\text{X}}_{\text{3}}{\text{X}}_{\text{4}}\;-\;0.013{\text{X}}_{\text{1}}{}^{\text{2}}\;+\;0.014{\text{X}}_{\text{2}}{}^{\text{2}}\;-\;0.022{\text{X}}_{\text{3}}{}^{\text{2}}\;+\;0.05{\text{X}}_{\text{4}}{}^{\text{2}} \end{array}$$ (7)

$$\begin{array}{l} R2\;=142.20\;-\;29.66{\text{X}}_{\text{1}}\;-58.10{\text{X}}_{\text{2}}\;-\;26.90{\text{X}}_{\text{3}}\;+\;39.40{\text{X}}_{\text{4}}\;+\;8.55{\text{X}}_{\text{1}}{\text{X}}_{\text{2}}\;+\;12.34{\text{X}}_{\text{1}}{\text{X}}_{\text{3}}\;+\;17.28{\text{X}}_{\text{1}}{\text{X}}_{\text{4}}\;-\\ 36.45{\text{X}}_{\text{2}}{\text{X}}_{\text{3}}\;-\;62.40{\text{X}}_{\text{2}}{\text{X}}_{\text{4}}\;-\;9.11{\text{X}}_{\text{3}}{\text{X}}_{\text{4}}\;-\;36.68{\text{X}}_{\text{1}}{}^{\text{2}}\;-3.09{\text{X}}_{\text{2}}{}^{\text{2}}\;-\;7.04{\text{X}}_{\text{3}}{}^{\text{2}}\;+\;34.00{\text{X}}_{\text{4}}{}^{\text{2}} \end{array}$$ (8)

$$\begin{array}{l} R3=34.51\;+\;0.74{\text{X}}_{\text{1}}\;+\;1.84{\text{X}}_{\text{2}}\;-\;4.11{\text{X}}_{\text{3}}\;+\;2.88{\text{X}}_{\text{4}}\;+\;6.51{\text{X}}_{\text{1}}{\text{X}}_{\text{2}}\;-\;3.39{\text{X}}_{\text{1}}{\text{X}}_{\text{3}}\;+\;1.97{\text{X}}_{\text{1}}{\text{X}}_{\text{4}}\;+\\ 2.83{\text{X}}_{\text{2}}{\text{X}}_{\text{3}}\;-\;1.18{\text{X}}_{\text{2}}{\text{X}}_{\text{4}}\;-\;7.32{\text{X}}_{\text{3}}{\text{X}}_{\text{4}}\;-\;1.62{\text{X}}_{\text{1}}{}^{\text{2}}\;-\;5.68{\;\text{X}}_{\text{2}}{}^{2}\;-\;5.83{\text{X}}_{\text{3}}{}^{\text{2}}\;-\;3.85{\text{X}}_{\text{4}}{}^{\text{2}} \end{array}$$ (9)

**Table 3:** Experimental matrix for BBD using R software package and DOE for medium components with actual and predicted responses (R1, R2, and R3).

Run No.	X_1_ Dye (ppm)		X_2_ CuSO_4_(mM)		X_3_ pH		X_4_ (Inoculum Size) (v/v %)		R1			R2			R3		
	Actual	Coded	Actual	Coded	Actual	Coded	Actual	Coded	Actual	Predic-ted By R Package	Predic-ted By DOE	Actual	Predic-ted By R Package	Predic-ted By DOE	Actual	Predic-ted By R Package	Predic-ted By DOE
**1**	20	-1	0.6	0	7.5	0	6	1	0.199	0.209	0.209	192.21	191.29	191.29	29.83	29.2	29.2
**2**	200	1	1	1	7.5	0	4	0	0.084	0.081	0.081	39.25	23.21	23.21	34.05	36.3	36.3
**3**	110	0	1	1	6	-1	4	0	0.113	0.116	0.116	134.56	137.31	137.31	29.08	26.12	26.12
**4**	200	1	0.6	0	7.5	0	2	-1	0.064	0.058	0.058	42.42	53.17	53.17	25.06	24.93	24.93
**5**	200	1	0.6	0	7.5	0	6	1	0.24	0.239	0.239	154.29	166.54	166.54	35.42	34.63	34.63
**6**	110	0	0.6	0	7.5	0	4	0	0.148	0.146	0.143	139.2	142.2	135.44	34.46	34.51	31.32
**7**	110	0	1	1	7.5	0	6	1	0.076	0.076	0.076	80.88	92.01	92.01	28.65	28.52	28.52
**8**	110	0	0.6	0	9	1	6	1	0.214	0.218	0.218	179.25	172.54	172.54	16.69	16.27	16.27
**9**	110	0	0.6	0	6	-1	2	-1	0.179	0.179	0.179	160.75	147.54	147.54	16.82	18.73	18.73
**10**	110	0	0.6	0	9	1	2	-1	0.088	0.087	0.087	112.68	111.97	111.97	24.9	25.15	25.15
**11**	110	0	1	1	9	1	4	0	0.003	0.007	0.007	9.87	10.62	10.61	23.18	23.55	23.55
**12**	110	0	0.2	-1	9	1	4	0	0.223	0.225	0.225	192.63	199.71	199.71	12	14.21	14.21
**13**	200	1	0.2	-1	7.5	0	4	0	0.124	0.143	0.143	137.02	122.31	122.31	20.02	19.6	19.6
**14**	110	0	0.2	-1	7.5	0	2	-1	0.156	0.146	0.146	130.45	129.41	129.41	19.7	19.07	19.07
**15**	110	0	1	1	7.5	0	2	-1	0.183	0.193	0.193	131.39	138.01	138.01	26.58	25.12	25.12
**16**	110	0	0.2	-1	6	-1	4	0	0.201	0.201	0.201	171.53	180.61	180.61	29.21	28.09	28.09
**17**	20	-1	1	1	7.5	0	4	0	0.075	0.06	0.06	70.64	65.43	65.43	19.88	21.8	21.8
**18**	20	-1	0.2	-1	7.5	0	4	0	0.294	0.301	0.301	202.61	198.73	198.73	31.88	31.13	31.13
**19**	110	0	0.6	0	7.5	0	4	0	0.148	0.146	0.143	139.2	142.2	135.44	34.46	34.51	31.32
**20**	200	1	0.6	0	9	1	4	0	0.061	0.055	0.055	55.62	54.25	54.25	21.59	20.29	20.29
**21**	110	0	0.6	0	7.5	0	4	0	0.148	0.146	0.143	139.2	142.2	135.44	34.46	34.51	31.32
**22**	110	0	0.6	0	7.5	0	4	0	0.138	0.146	0.143	154.2	142.2	135.44	34.52	34.51	31.32
**23**	110	0	0.6	0	7.5	0	4	0	0.148	0.146	0.143	139.2	142.2	135.44	34.65	34.51	31.32
**24**	110	0	0.2	-1	7.5	0	6	1	0.447	0.427	0.427	329.55	333.01	333.01	26.49	27.19	27.19
**25**	200	1	0.6	0	6	-1	4	0	0.101	0.096	0.096	74.24	83.37	83.37	34.93	35.3	35.3
**26**	20	-1	0.6	0	6	-1	4	0	0.17	0.166	0.166	155.91	167.37	167.39	26.48	27.02	27.02
**27**	20	-1	0.6	0	7.5	0	2	-1	0.221	0.226	0.226	149.48	147.06	147.06	27.34	27.38	27.38
**28**	20	-1	0.6	0	9	1	4	0	0.127	0.122	0.122	87.94	88.9	88.9	26.72	25.59	25.59
**29**	110	0	0.6	0	6	-1	6	1	0.207	0.212	0.212	263.78	244.57	244.57	37.88	39.13	39.13

**Table 4:** Regression analysis of response R1, R2, and R3 by BBD of process parameters using R software.

	R1				R2				R3			
	Estimate	Std. Error	t value	Pr(>|t|)	Estimate	Std. Error	t value	Pr(>|t|)	Estimate	Std. Error	t value	Pr(>|t|)
**Intercept**	0.146	0.0048769	29.9368	4.295E-14 ***	142.2	5.4719	25.9871	3.013E-13 ***	34.51	0.73201	47.144	< 2.2E-16***
**X**_**1**_	-0.0343333	0.003148	-10.9062	3.157E-08 ***	-29.6625	3.5321	-8.3979	7.748E-07 ***	0.745	0.47251	1.5767	0.1371915
**X**_**2**_	-0.0759167	0.003148	-24.1155	8.396E-13 ***	-58.1	3.5321	-16.449	1.494E-10 ***	1.84333	0.47251	3.9011	0.0015982**
**X**_**3**_	-0.02125	0.003148	-6.7502	9.316E-06 ***	-26.8983	3.5321	-7.6153	2.419E-06 ***	-4.11	0.47251	-8.6982	5.10E-07***
**X**_**4**_	0.041	0.003148	13.0239	3.247E-09 ***	39.3992	3.5321	11.1545	2.375E-08 ***	2.88	0.47251	6.0951	2.77E-05***
**X**_**1**_**X**_**2**_	0.04475	0.0054526	8.2071	1.016E-06 ***	8.55	6.1178	1.3976	0.184	6.5075	0.81842	7.9513	1.47E-06***
**X**_**1**_**X**_**3**_	0.00075	0.0054526	0.1375	0.8925551	12.3375	6.1178	2.0167	0.06333	-3.395	0.81842	-4.1483	0.0009849***
**X**_**1**_**X**_**4**_	0.0495	0.0054526	9.0783	3.052E-07 ***	17.285	6.1178	2.8254	0.01349 *	1.9675	0.81842	2.404	0.0306312*
**X**_**2**_**X**_**3**_	-0.033	0.0054526	-6.0522	2.976E-05 ***	-36.4475	6.1178	-5.9576	3.501E-05 ***	2.8275	0.81842	3.4548	0.0038672**
**X**_**2**_**X**_**4**_	-0.0995	0.0054526	-18.2482	3.711E-11 ***	-62.4025	6.1178	-10.2001	7.304E-08 ***	-1.18	0.81842	-1.4418	0.1713506
**X**_**3**_**X**_**4**_	0.0245	0.0054526	4.4933	0.0005057 ***	-9.115	6.1178	-1.4899	0.15843	-7.3175	0.81842	-8.9411	3.67E-07***
**X**_**1**_^**2**^	-0.0134167	0.0042818	-3.1334	0.0073294 **	-36.6858	4.8042	-7.6362	2.344E-06 ***	-1.62083	0.64269	-2.522	0.0244076*
**X**_**2**_^**2**^	0.0139583	0.0042818	3.2599	0.0056992 **	-3.0921	4.8042	-0.6436	0.53022	-5.68083	0.64269	-8.8392	4.21E-07***
**X**_**3**_^**2**^	-0.0225417	0.0042818	-5.2645	0.0001196 ***	-7.0446	4.8042	-1.4663	0.16466	-5.83583	0.64269	-9.0804	3.04E-07***
**X**_**4**_^**2**^	0.0508333	0.0042818	11.8719	1.073E-08 ***	34.0017	4.8042	7.0775	5.529E-06 ***	-3.85083	0.64269	-5.9918	3.30E-05***
	(Significant. codes: 0—‘***’, 0.001—‘**’, 0.01—‘*’)											

The Eqs 7–9 obtained from the experiments were tested for R^2^, being a measure of the goodness of fit for the regression model was 0.9917, 0.9831 and 0.9703 for responses R1, R2 and R3 respectively. The adjusted R^2^ value for R1, R2, and R3 were 0.9835, 0.9662 and 0.9405 respectively. The R^2^ and adjusted R^2^ values for all responses are close to 1.0 indicated that the equations is highly reliable and increase strength of the relationship between model and response.

The ANOVA model for all responses was significant and the model F-value of R1, R2 and R3 were 120.13, 58.22 and 32.63 respectively. The F values of models were found to be high which suggests that data not supported by the null hypothesis. The p-value <0.05 indicates a statistically significant model. The linear, square and interactions terms for all responses were significant (p ≤ 0.001) (Table 5). Thus, the overall model of all three responses has been found to be precisely accurate, significant, and reproducible. To the best of our knowledge, this is the first attempt to optimize the multiple responses using R software package parallelly.

**Table 5:** ANOVA of responses R1, R2, and R3 for BBD of process parameters using R software.

	R1					R2					R3				
	Df	Sum Sq	Mean Sq	F value	Pr(>F)	Df	Sum Sq	Mean Sq	F value	Pr(>F)	Df	Sum Sq	Mean Sq	F value	Pr(>F)
**Model**	14	0.2	0.01428	120.12	4.29E-12***	14	122024.62	8716.044	58.22	6.14E-10***	14	1224.0102	87.4293	32.63	3.01E-08***
**Linear**	4	0.108896	0.027224	228.92	1.38E-12***	4	78375	19593.9	130.88	6.35E-11***	4	349.67	87.418	32.63	5.86E-07***
**Interaction**	6	0.064172	0.0106953	89.93	2.27E-10***	6	23319	3886.4	25.96	8.04E-07***	6	482.71	80.452	30.03	3.20E-07***
**Square**	4	0.026933	0.0067333	56.62	1.72E-08***	4	20331	5082.6	33.95	4.57E-07***	4	391.63	97.907	36.54	2.88E-07***
**Residuals**	14	0.001665	0.0001189			14	2096	149.7			14	37.51	2.679		
**Lack of fit**	10	0.001585	0.0001585	7.92	0.03043	10	1916	191.6	4.26	0.08763	10	37.48	3.748	551.20	7.87E-06***
**Pure error**	4	0.00008	0.00002			4	180	45			4	0.03	0.007		
**Total**	28	0.201665				28	124120.57				28	1261.5192			
	(Significant codes: 0 –’***’, 0.001 –’**’, 0.01 –’*’)														

It is well understood from Table 3, that the prediction of R software’s package is more near to actual values than Design-Expert for various responses (i.e., run no: 06, 19, 21, 22, 23) which depends on the degree of protection against biases in model construction [66]. Moreover, in all 29 runs, the average meant difference between actual and predicted values of responses R1, R2 and R3, were least (0.00024, 0.00034 and 0.00206 respectively) using R software, whereas similar values were found higher with Design-Expert, i.e., 0.00075, 1.16551 and 0.55206 for R1, R2, and R3 respectively. Thus, results indicate R offer a higher level of protection against biases than Design-Expert.

### Comparison of R and DOE software package model

#### Predictive potentiality

The predictive capabilities of R software package and DOE models were compared using the R^2^, Root Means Square Error (RMSE), and Mean Absolute Error (MAE) values (Table 6). The predictive values of R1, R2, and R3 by R software are more suitable and justifiable than the predictive responses values of DOE.

**Table 6:** Comparison of predictive capability between R Software package and DOE.

Parameters	R Software Package			DOE		
	R1	R2	R3	R1	R2	R3
**R^2^**	0.9917	0.9831	0.9703	0.9893	0.9822	0.8442
**RMSE**	0.0076	8.5014	1.1373	0.0086	8.7073	2.5739
**MAE**	0.0055	6.7181	0.8269	0.0065	6.9277	1.5757

The R^2^, MAE and RMSE values predicted by R software package model proved to be superior over DOE for all responses. In contrast to DOE data, R package data showed the lower standard error of the regression line which represents a quick approximation of 95% prediction interval (S1 and S2 Tables).

#### Sensitivity analysis

**Fig 3:**
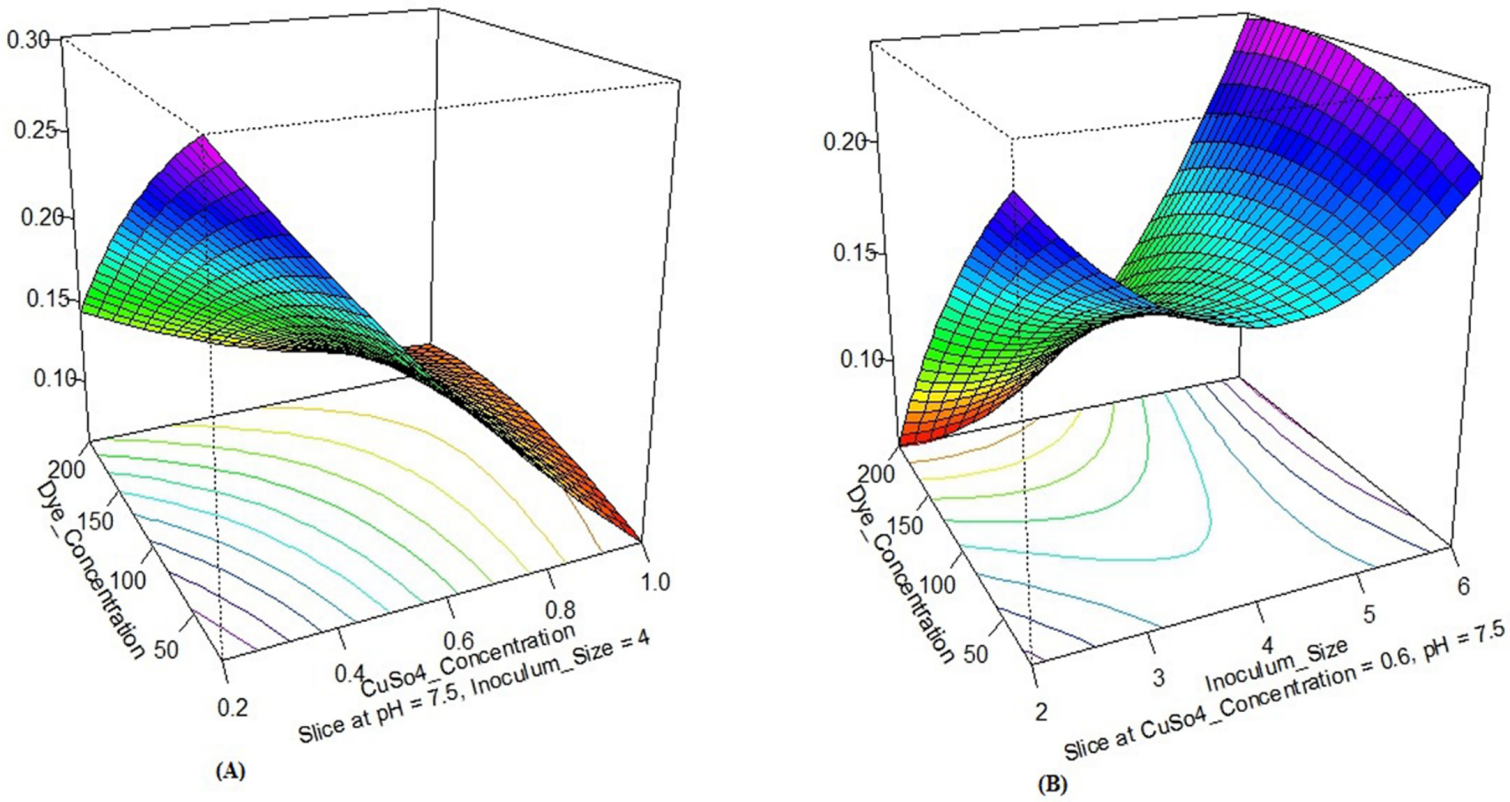
Contour plots show the response surface effect of interaction on the growth of isolates. (A) X_1_ with X_2_, and (B) X_1_ with X_4_.

**Fig 4:**
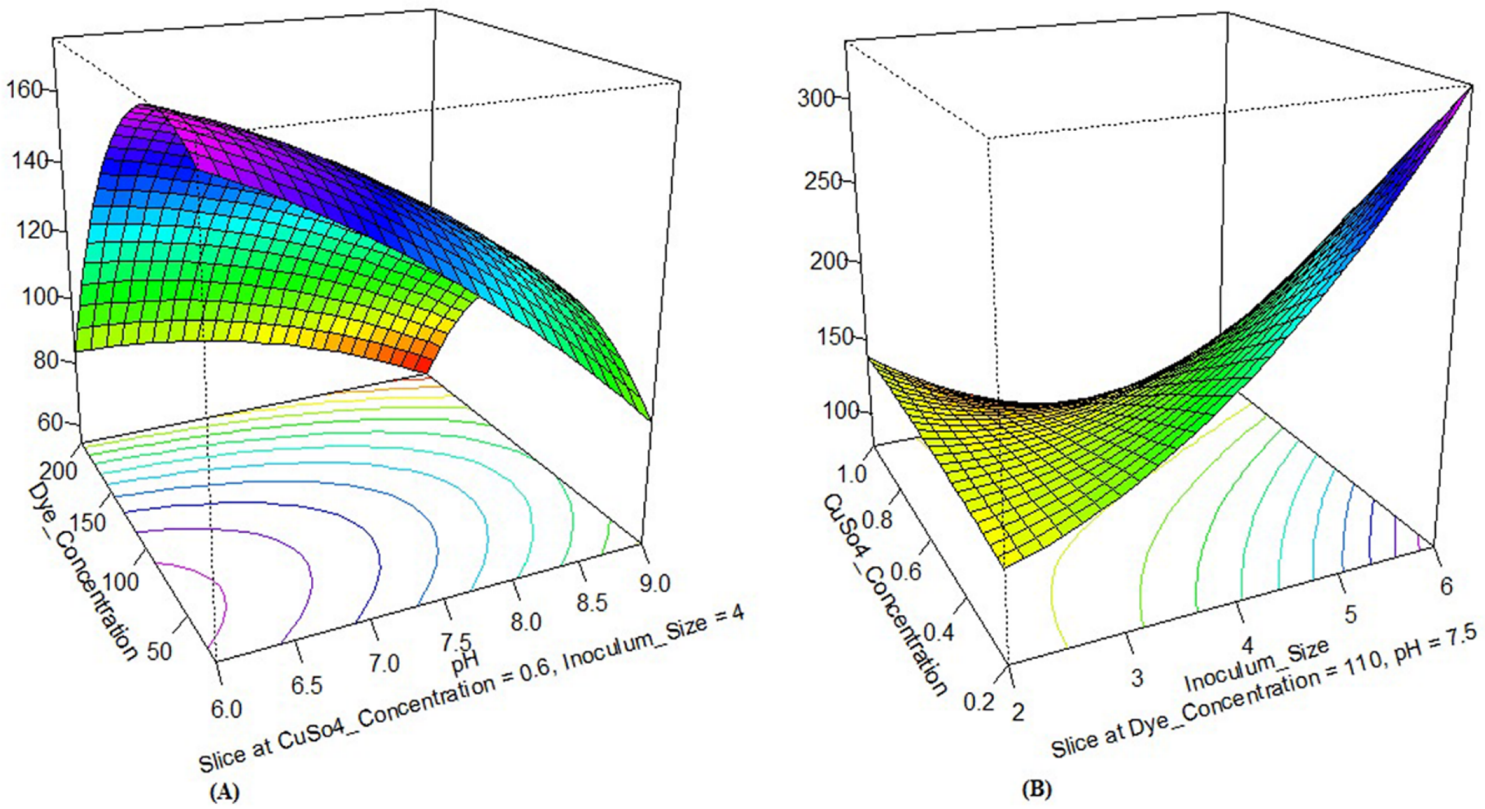
Contour plots show the response surface effect of interaction on laccase activity. (A) X_1_ with X_3_, and (B) X_2_ with X_4_.

**Fig 5:**
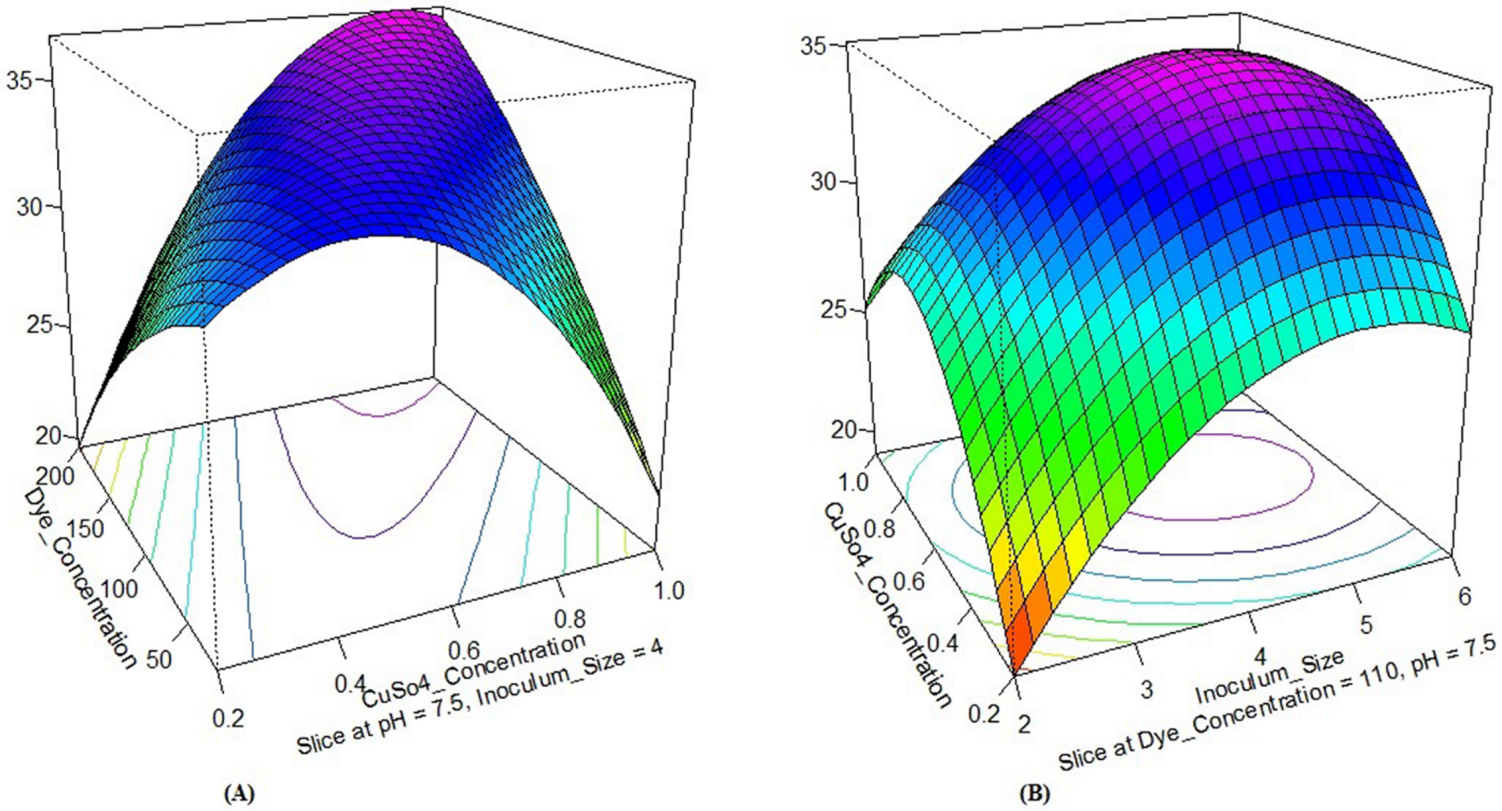
Contour plots show the response surface effect of interaction on dye decolorization. (A) X_1_ with X_3_, and (B) X_2_ with X_4_.

**Fig 6:**
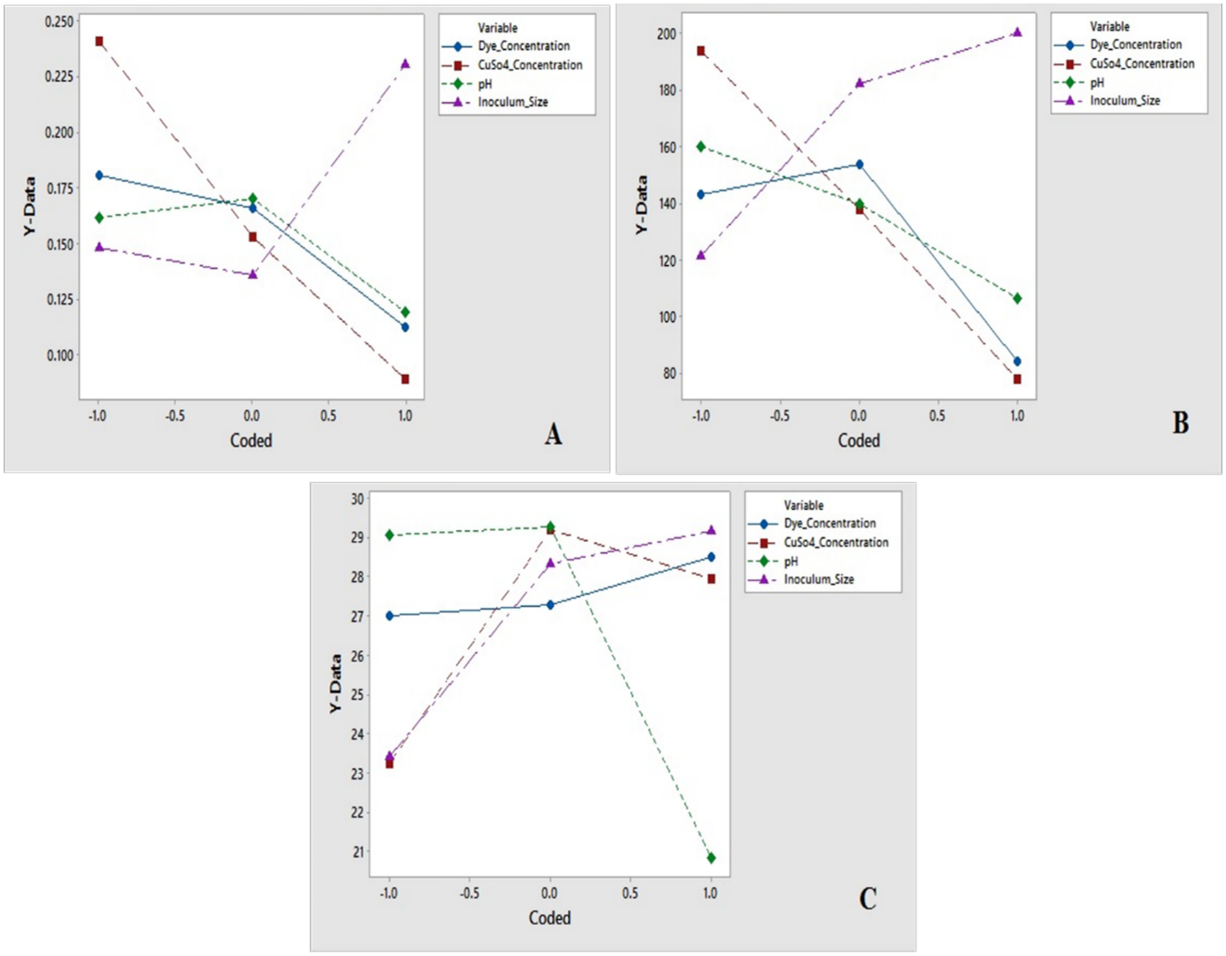
Sensitivity analysis of response. (A) For R1, (B) For R2, and (C) For R3.

The effect of individual medium components and their interactions can be studied in a superior way using R software package through DOE linking. Coefficients of R software package and DOE have attributed a direct measure of the contribution of various medium components in the system. In this study, a sensitivity analysis performed for determining the effectiveness of a parameter by the constructed R software package model using ‘Perturb’ method [67]. The constructed R software package model perform sensitivity analysis using ‘Perturn’ method for determining the effectiveness of a parameter. Response surface effects of interaction between X_1_ and X_2_; X_1_ and X_3_ as well as X_2_ and X_4_ for all three responses were also significant which shown in Figs 3, 4 and 5. Fig 6 shows the sensitivity analysis of R software package demonstrating the rate of response and it is varied with a change in the input variable.

Out of four variables for response R1 and R2, X_4_ had the highest coefficient value; signifying inoculum size as the most influencing variable for the growth of isolate. X_1_X_4_ had maximum coefficient value compared to other interactions, and X_4_^2^ had maximum coefficient value compared to other squared; suggesting their significant effect on growth promotion of isolate and enhance the laccase activity (Table 4).

Similarly, for R3, out of four variables, X_4_ had the highest coefficient value, and X_2_ had second highest coefficient value; signifying inoculum size and copper sulphate concentration is the most influencing variables for the dye decolorization respectively. X_1_X_2_ had maximum coefficient value compared to other interactions; suggesting their significant effect on enhancing dye decolorization.

#### Validation of model

Under the optimal conditions, 0.454 ± 0.004 growth (A_620_nm) with 336 ± 4.32 U/mL laccase activity and 51.33 ± 5.50% decolorization of disperse blue DBR was experimentally obtained by isolate (Table 7), which is in good agreement with the decolorization predicted by the model. Decolorization using 336 ± 4.32 U/mL of the enzyme is less than the 285.33 ± 5.03 U/mL of the enzyme. It is due to the acidic pH 6.0 of the media. The disperse blue DBR readily decolorized in acidic pH than alkali pH [68,69]. Secondly, CuSO_4_ and its interaction with dye (X_1_X_2_) positively affecting in the decolorization process.

**Table 7:** Optimized medium component for validation of a model for responses R1, R2, and R3.

Run No.	X_1_ (ppm)	X_2_ (mM)	X_3_	X_4_ (% v/v)	R1	R2 (U/mL)	R3 (%)
24	110	0.2	7.5	6	0.454 ± 0.004	336 ± 4.32	31.33 ± 3.21
29	110	0.6	6	6	0.280 ± 0.001	285.33 ± 5.03	51.33 ± 5.50

### Toxicity evaluation of decolorized dye

*In vitro* cytotoxicity test is the principle property of any compound surrounded by living cells or organisms. The result of cytotoxicity assay indicates the laccase decolorized dye sample was less toxic than untreated control dye in both tested cell lines. Laccase treated sample indicate the only 12.14 ± 0.52% toxicity on MCF-7 cell line, whereas untreated dye sample revealed the 49.41 ± 0.49% (Table 8). The normal human lung epithelial cell line (L132) by treated and untreated dye samples indicated less cytotoxic difference compared to MCF-7 cell line. Cell viability of the untreated sample in both cell lines is due to the autolysis of dye during vigorous shaking for a prolonged time. The degradation and decolorization of synthetic azo dyes by laccase enzymes depends on a formation of a nonspecific free radical mechanism to form nontoxic phenolic compounds and avoid the formation of toxic aromatic compounds.

**Table 8:** Toxicity evaluation of decolorized dye on human cell lines.

Sr. No.	Cell line	Parameter	Laccase treated	Untreated sample
**1**	MCF-7	% Cell viability	87.22 ± 0.48	50.41 ± 0.64
		% Cytotoxicity	12.14 ± 0.52	49.41 ± 0.49
**2**	L132	% Cell viability	79.60 ± 0.45	63.49 ± 0.08
		% Cytotoxicity	20.10 ± 0.73	36.21 ± 0.30

However, this is the preliminary study which is helpful in designing the prototype of bioprocess optimization for decolorization of the dye. The in-situ or ex-situ large scale optimization may be required to establish the “R” based scale up process for bioremediation purpose.

## Conclusion

This first report of the simultaneous optimization of factors affecting multiple responses- the growth of isolate, laccase, production and dye decolorization using R software and DOE. The thermophilic bacterium, *Bacillus licheniformis* U1 was found suitable for the bulk production of thermophilic laccase. The optimization of media components using BBD, 1.80 fold rises in laccase production had achieved along with 2.24 fold enhancement in dye decolorization. Laccase treated sample was found less toxic on both the cell line. So, the detoxification study suggests the impending application of enzyme to lessen the toxicity of textile effluents. Inoculum size (X_4_) was found to have the highest effects on all the responses. Moreover, inoculum size interaction with dye concentration (X_1_X_4_) shown the positive impact on growth promotion of bacterium. Copper sulphate was surfaced out as a crucial component of dye decolorization process. The average mean difference between actual and predicted values of all BBD generated runs was negligible in R software. So, R offers the better protection against biases than DOE. Furthermore, the ANOVA and regression analysis of all responses using R software was found more trustworthy than the DOE. The R2, RMSE, and MAE calculated using R software suggest the tremendous predictive potentiality. However, further reactor based process scale-up is necessary for actual industrial applications. Thus, the study would undoubtedly embolden the scientific community to extensively exploit the significant features of R software package over DOE analyses.

## Supporting information

S1 Table Regression analysis of response R1, R2, and R3 by BBD of process parameters using DOE. (DOCX)

S2 Table ANOVA of responses R1, R2, and R3 for BBD of process parameters using DOE. (DOCX)
