# Supplementary material for: R software package based statistical optimization of process components to simultaneously enhance the bacterial growth, laccase production and textile dye decolorization with cytotoxicity study
Source: PLoS One. 2018 May 2;13(5):e0195795. doi: 10.1371/journal.pone.0195795 (PMC5931462; doi:10.1371/journal.pone.0195795)
Supplement: S1 Table — (DOCX) [file pone.0195795.s001.docx]

**S1 Table. Regression analysis of response R1, R2, and R3 by BBD of process parameters using DOE.**

|  | **R1** | | | **R2** | | | **R3** | | |
| --- | --- | --- | --- | --- | --- | --- | --- | --- | --- |
|  | **Estimate** | **Std. Error** | **Pr(>\|t\|)** | **Estimate** | **Std. Error** | **Pr(>\|t\|)** | **Estimate** | **Std. Error** | **Pr(>\|t\|)** |
| **(Intercept)** | 0.1426 | 0.005565 | 2.60E-11*** | 135.44 | 5.604467 | 8.58E-10*** | 31.318 | 1.65669 | 0.001591** |
| **X_1_** | -0.03433 | 0.003592 | 1.63E-07*** | -29.6625 | 3.617668 | 1.03E-06*** | 0.745 | 1.069389 | 0.497421 |
| **X_2_** | -0.07592 | 0.003592 | 5.09E-12*** | -58.1 | 3.617668 | 2.06E-10*** | 1.843333 | 1.069389 | 0.106759 |
| **X_3_** | -0.02125 | 0.003592 | 3.77E-05*** | -26.8983 | 3.617668 | 3.18E-06*** | -4.11 | 1.069389 | 0.001791** |
| **X_4_** | 0.041 | 0.003592 | 1.78E-08*** | 39.39917 | 3.617668 | 3.21E-08*** | 2.88 | 1.069389 | 0.01749 |
| **X_1_X_2_** | 0.04475 | 0.006222 | 4.62E-06*** | 8.55 | 6.265985 | 0.193939 | 6.5075 | 1.852236 | 0.003443** |
| **X_1_X_3_** | 0.00075 | 0.006222 | 0.905772 | 12.3375 | 6.265985 | 0.069079 | -3.395 | 1.852236 | 0.088163 |
| **X_1_X_4_** | 0.0495 | 0.006222 | 1.46E-06*** | 17.285 | 6.265985 | 0.015385 | 1.9675 | 1.852236 | 0.306112 |
| **X_2_X_3_** | -0.033 | 0.006222 | 0.000111*** | -36.4475 | 6.265985 | 4.47E-05*** | 2.8275 | 1.852236 | 0.149149 |
| **X_2_X_4_** | -0.0995 | 0.006222 | 2.18E-10*** | -62.4025 | 6.265985 | 9.83E-08*** | -1.18 | 1.852236 | 0.53436 |
| **X_3_X_4_** | 0.0245 | 0.006222 | 0.001488** | -9.115 | 6.265985 | 0.16781 | -7.3175 | 1.852236 | 0.00145** |
| **X_1_^2^** | -0.01172 | 0.004886 | 0.030993 | -33.3058 | 4.920561 | 9.04E-06*** | -0.02483 | 1.454526 | 0.986619 |
| **X_2_^2^** | 0.015658 | 0.004886 | 0.006362** | 0.287917 | 4.920561 | 0.954167 | -4.08483 | 1.454526 | 0.013949 |
| **X_3_^2^** | -0.02084 | 0.004886 | 0.000784*** | -3.66458 | 4.920561 | 0.468742 | -4.23983 | 1.454526 | 0.011304 |
| **X_4_^2^** | 0.052533 | 0.004886 | 3.78E-08*** | 37.38167 | 4.920561 | 2.49E-06*** | -2.25483 | 1.454526 | 0.143395 |
|  | (Significant. codes: 0 - '***', 0.001 - ‘**', 0.01 - '*') | | | | | | | | |
