# Supplementary material for: R software package based statistical optimization of process components to simultaneously enhance the bacterial growth, laccase production and textile dye decolorization with cytotoxicity study
Source: PLoS One. 2018 May 2;13(5):e0195795. doi: 10.1371/journal.pone.0195795 (PMC5931462; doi:10.1371/journal.pone.0195795)
Supplement: S2 Table — (DOCX) [file pone.0195795.s002.docx]

**S2 Table. ANOVA of responses R1, R2, and R3 for BBD of process parameters using DOE**.

|  | **R1** | | | | | **R2** | | | | | **R3** | | | | |
| --- | --- | --- | --- | --- | --- | --- | --- | --- | --- | --- | --- | --- | --- | --- | --- |
|  | **Df** | **Sum Sq** | **Mean Sq** | **F value** | **Pr(>F)** | **Df** | **Sum Sq** | **Mean Sq** | **F value** | **Pr(>F)** | **Df** | **Sum Sq** | **Mean Sq** | **F value** | **Pr(>F)** |
| **Model** | 14 | 0.20045 | 0.014318 | 92.45 | 2.60E-11*** | 14 | 121855.08 | 8703.934 | 55.42 | 8.58E-10*** | 14 | 1041.7845 | 74.41318 | 5.42 | 0.0015908** |
| **Linear** | 4 | 0.108896 | 0.108896 | 703.17 | 3.79E-05*** | 4 | 78375.46 | 78375.46 | 499.05 | 4.24E-06*** | 4 | 349.67283 | 349.67283 | 25.48 | 0.623461 |
| **Interaction** | 6 | 0.064172 | 0.064172 | 414.37 | 9.07E-01*** | 6 | 23318.65 | 23318.65 | 148.47 | 0.4462578 | 6 | 482.7104 | 482.7104 | 35.17 | 1.0826768 |
| **Square** | 4 | 0.0232 | 0.0232 | 149.80 | 0.788122 | 4 | 16347.11 | 16347.11 | 104.09 | 1.42E+00 | 4 | 257.81799 | 257.81799 | 18.79 | 1.1552665 |
| **Residuals** | 14 | 0.002168 | 0.000155 |  |  | 14 | 2198.704 | 157.0503 |  |  | 14 | 192.1235 | 13.72311 |  |  |
| **Lack of fit** | 10 | 0.001585 | 0.000158 | 1.087 | 0.510501 | 10 | 1915.952 | 191.5952 | 2.71 | 0.174483 | 10 | 37.4818 | 3.74818 | 0.09 | 0.99858 |
| **Pure error** | 4 | 0.000583 | 0.000146 |  |  | 4 | 282.752 | 70.688 |  |  | 4 | 154.6417 | 38.66042 |  |  |
| **Total** | 28 | 0.202623 |  |  |  | 28 | 124053.8 |  |  |  | 28 | 1233.908 |  |  |  |
|  | (Significant codes: 0 – ‘***’, 0.001 – “**’, 0.01 – “*’) | | | | | | | | | | | | | | |
